# Supplementary material for: Could traces of fluoroquinolones in food induce ciprofloxacin resistance in Escherichia coli and Klebsiella pneumoniae? An in vivo study in Galleria mellonella with important implications for maximum residue limits in food
Source: Microbiol Spectr. 2024 Apr 30;12(6):e03595-23. doi: 10.1128/spectrum.03595-23 (PMC11237748; doi:10.1128/spectrum.03595-23)
Supplement: Supplementary material — Fig. S1 to S6; Tables S1 and S2. [file spectrum.03595-23-s0001.docx]

**Supplementary file**

[SFigure 1. Scatter bar plot of number of colonies of each species isolated on selective agar plates without ciprofloxacin per day for the single dose ciprofloxacin experiments 2](#_Toc159936705)

[SFigure 2. Scatter bar plot of number of colonies of each species isolated on selective agar plates without ciprofloxacin per day for the daily dose experiments 3](#_Toc159936706)

[SFigure 3. Mortality of Galleria mellonella larvae injected with E. coli, K. pneumoniae M14827 and K. pneumoniae M17125 4](#_Toc159936707)

[SFigure 4. Ciprofloxacin MICs of bacterial colonies of *E. coli*, *K. pneumoniae* M14827 and *K. pneumoniae* M17125 taken from plates ***without*** ciprofloxacin (0.125 μg/ml) 5](#_Toc159936708)

[SFigure 5. Ciprofloxacin MICs of bacterial colonies of *K. pneumoniae* M14827 after exposure to **daily** ciprofloxacin 6](#_Toc159936709)

[SFigure 6. Schematic diagram of the genomic regions comprising (A) *ramR* 7](#_Toc159936710)

[and *ramA* and (B) *rfr2* and *oqxB20* of *K. pneumoniae* M14827 (Produced in CLC Genomics Workbench 20). 7](#_Toc159936711)

[STable 1. List of all mutations detected in WGS for *K. pneumoniae* M14827 8](#_Toc159936712)

[STable 2. List of all mutations detected in WGS for *K. pneumoniae* M17125 9](#_Toc159936713)

SFigure 1. Scatter bar plot of number of colonies of each species isolated on selective agar plates without ciprofloxacin per day for the single dose ciprofloxacin experiments (bar charts represent mean number of colonies and standard deviation)

SFigure 2. Scatter bar plot of number of colonies of each species isolated on selective agar plates without ciprofloxacin per day for the daily dose experiments (bar charts represent mean number of colonies and standard deviation)

SFigure 3. Mortality of Galleria mellonella larvae injected with E. coli, K. pneumoniae M14827 and K. pneumoniae M17125 (* P<0.01). The larvae sacrificed daily for cultures are counted as dead on the day of sacrifice.

# SFigure 4. Ciprofloxacin MICs of bacterial colonies of *E. coli*, *K. pneumoniae* M14827 and *K. pneumoniae* M17125 taken from plates ***without*** ciprofloxacin (0.125 μg/ml)

SFigure 5. Ciprofloxacin MICs of bacterial colonies of *K. pneumoniae* M14827 after exposure to **daily** ciprofloxacin (ADI dose, 0.1xADI dose or control/PBS). Mean and SEM shown.

# SFigure 6. Schematic diagram of the genomic regions comprising (A) *ramR*

# and *ramA* and (B) *rfr2* and *oqxB20* of *K. pneumoniae* M14827 (Produced in CLC Genomics Workbench 20).

A





B


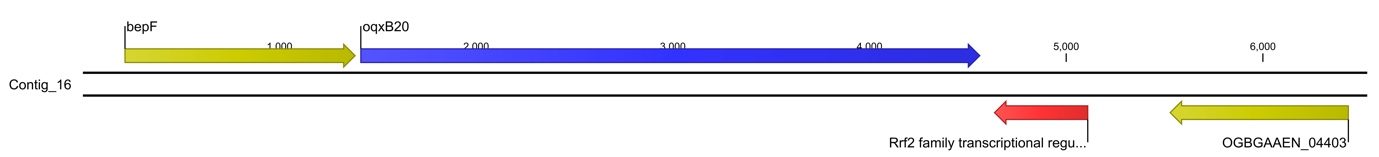


# STable 1. List of all mutations detected in WGS for *K. pneumoniae* M14827

#N/A Not Applicable or not detected

# STable 2. List of all mutations detected in WGS for *K. pneumoniae* M17125

| Overlapping annotations | M17125-1A_control_invivoD7 | KPZCip4Mic0.38/2 | KPN1in1000Q1 | KPN1in100Q2 | KPQ1_Base |
| --- | --- | --- | --- | --- | --- |
| CDS: FJNPIGNM_04598, Gene: FJNPIGNM_04598 | X:FJNPIGNM_04598:p.Leu82Pro | X:FJNPIGNM_04598:p.Leu82Pro | X:FJNPIGNM_04598:p.Leu82Pro | X:FJNPIGNM_04598:p.Leu82Pro | X:FJNPIGNM_04598:p.Leu82Pro |
| Gene: traI_1, CDS: traI_1 (Multifunctional conjugation protein TraI-size 5262 aa) | X:FJNPIGNM_04817:c.1233C>T X:FJNPIGNM_04817:c.1119T>C | X:FJNPIGNM_04817:c.1509G>A | X:FJNPIGNM_04817:c.1509G>A | #N/A | #N/A |
| Gene: FJNPIGNM_04824, CDS: FJNPIGNM_04824 | X:FJNPIGNM_04824:c.69C>T | X:FJNPIGNM_04824:c.69C>T | X:FJNPIGNM_04824:c.69C>T | X:FJNPIGNM_04824:c.69C>T | X:FJNPIGNM_04824:c.69C>T |
| Gene: traQ, CDS: traQ | X:FJNPIGNM_04825:c.82A>C | X:FJNPIGNM_04825:c.82A>C | X:FJNPIGNM_04825:c.82A>C | X:FJNPIGNM_04825:c.82A>C | X:FJNPIGNM_04825:c.82A>C |
| Gene: FJNPIGNM_04826, CDS: FJNPIGNM_04826 | X:FJNPIGNM_04826:c.579T>C | X:FJNPIGNM_04826:c.579T>C | X:FJNPIGNM_04826:c.579T>C | X:FJNPIGNM_04826:c.579T>C | X:FJNPIGNM_04826:c.579T>C |
| Gene: FJNPIGNM_04843, CDS: FJNPIGNM_04843 | X:FJNPIGNM_04843:c.828T>C X:FJNPIGNM_04843:c.810C>T X:FJNPIGNM_04843:c.790C>G X:FJNPIGNM_04843:c.543C>T X:FJNPIGNM_04843:c.318T>C | X:FJNPIGNM_04843:c.1080G>A X:FJNPIGNM_04843:c.543C>T | X:FJNPIGNM_04843:c.1080G>A | #N/A | X:FJNPIGNM_04843:c.1080G>A |
| Gene: FJNPIGNM_04844, CDS: FJNPIGNM_04844 | X:FJNPIGNM_04844:p.Ala15Val | X:FJNPIGNM_04844:p.Ala15Val | X:FJNPIGNM_04844:p.Ala15Val | X:FJNPIGNM_04844:p.Ala15Val | X:FJNPIGNM_04844:p.Ala15Val |
| Gene: FJNPIGNM_04845, CDS: FJNPIGNM_04845 | X:FJNPIGNM_04844:p.Ala15Val X:FJNPIGNM_04845:p.Ser198Pro X:FJNPIGNM_04845:c.466C>T X:FJNPIGNM_04845:p.Met147Arg X:FJNPIGNM_04845:c.435T>G X:FJNPIGNM_04845:p.His88Arg X:FJNPIGNM_04845:c.72A>G | X:FJNPIGNM_04845:p.His88Arg X:FJNPIGNM_04845:c.72A>G | X:FJNPIGNM_04845:p.His88Arg | X:FJNPIGNM_04845:p.Ser198Pro X:FJNPIGNM_04845:p.His88Arg X:FJNPIGNM_04845:c.72A>G | X:FJNPIGNM_04845:p.His88Arg X:FJNPIGNM_04845:c.72A>G |
| Gene: FJNPIGNM_04870, CDS: FJNPIGNM_04870 | #N/A | #NA | X:FJNPIGNM_04870:p.Arg96fs | X:FJNPIGNM_04870:p.Arg96fs | #N/A |

#N/A Not Applicable or not detected
